# A case report of spherical pneumonia caused by *Chlamydia psittaci* infection

**DOI:** 10.3389/fmed.2026.1841775

**Published:** 2026-06-08

**Authors:** Guodong Li, Xiangze Li, Qian Feng, Xiaodong Wang, Xingcai Zhang, Xue Liu

**Affiliations:** 1 Department of Pulmonary and Critical Care Medicine, Affiliated Hospital of Shandong University of Traditional Chinese Medicine, Jinan, China; 2 Department of Gastrointestinal Surgery, Shandong Provincial Third Hospital, Shandong University, Jinan, China; 3 Department of Pathology, Affiliated Hospital of Shandong University of Traditional Chinese Medicine, Jinan, China

**Keywords:** case report, *Chlamydia psittaci*, computed tomography, NGS, spherical pneumonia, tetracyclines

## Abstract

Psittacosis is a zoonotic infectious disease caused by *Chlamydia psittaci*, typically transmitted from birds to humans. Its clinical manifestations are often subtle and non-specific, making it prone to being misdiagnosed as common community-acquired pneumonia. Round pneumonia (or spherical pneumonia) refers to a lung infection that appears as a round or oval density on computed tomography (CT). It is uncommon in adults. Spherical pneumonia specifically caused by *Chlamydia psittaci* infection is particularly uncommon. Here we report a case of a 43-year-old male who was admitted due to a 4-day history of fever. CT imaging revealed a solitary, round high-density shadow approximately 4 cm in diameter in the right lower lung, with clear margins and surrounding ground-glass opacity. The patient reported direct contact with ornamental parrots at a zoo 10 days before the onset of symptoms. The diagnosis of spherical pneumonia caused by *Chlamydia psittaci* infection was confirmed by targeted next-generation sequencing (tNGS). The patient’s symptoms resolved following treatment with minocycline, and a follow-up CT scan 32 days later showed near-complete resolution of the lesion. This case demonstrates that *Chlamydia psittaci* can also produce a typical spherical lesion, thereby broadening the recognized imaging spectrum of this pathogen. When clinicians encounter a patient with imaging findings suggestive of spherical pneumonia and a history of bird exposure, *Chlamydia psittaci* infection should be suspected, especially when accompanied by extrapulmonary symptoms such as headache, myalgia, or hepatosplenomegaly, necessitating a comprehensive assessment that integrates epidemiological history and molecular diagnostic methods.

## 1 Introduction

Psittacosis is a zoonotic disease caused by *Chlamydia psittaci*. Humans can be infected by inhaling aerosols from the excreta of infected birds (1). Non-avian animals [such as turkeys, ducks, rodents, cattle, etc. (2)] can also carry the bacterium. Human-to-human transmission is rare but remains possible (3). Psittacosis most commonly presents as human atypical pneumonia, representing a rare cause of community-acquired pneumonia (CAP). Studies indicate that among severe pneumonia patients admitted to the ICU, the infection rate of *Chlamydia psittaci*can be as high as 8% (4). Symptoms in severe cases are often atypical; beyond severe pneumonia, patients may exhibit systemic toxic symptoms involving multiple organ systems, such as pericarditis, myocarditis, acute kidney injury, pancreatitis, jaundice, and gastrointestinal bleeding (5). Disease progression is closely associated with patient age, underlying conditions (particularly cardiopulmonary diseases), smoking history, nutritional status, and the timeliness of diagnosis and treatment (6). Its imaging manifestations typically include multi-lobar consolidations, ground-glass opacities, pleural effusion, halo sign, reversed halo sign. Cavitary lesions and pulmonary nodules with a halo sign have also been reported (7, 8). In contrast, spherical pneumonia is rarely documented (9). Spherical pneumonia is an imaging manifestation of pulmonary lesions, frequently appearing as round or round-like consolidation shadows on chest imaging, with limited reports in the literature (10, 11). It is mostly caused by acute inflammation from bacteria or viruses, primarily *Streptococcus pneumoniae* and *Staphylococcus* (12). Related studies indicate that the common pathogens of spherical pneumonia are similar to those of community-acquired pneumonia, but it is seldom associated with psittacosis pneumonia. Spherical pneumonia is easily misdiagnosed as a lung tumor or tuberculoma. Therefore, awareness of this condition must be enhanced.

## 2 Case report

A 43-year-old male was admitted to the respiratory department of our hospital due to a 4-day history of high fever. He presented with accompanying fatigue and myalgia, but reported no significant cough, sputum production, chest tightness, or shortness of breath. The patient had direct contact with parrots at a zoo approximately 10 days prior to symptom onset. The patient, a police officer, had no history of smoking or exposure to harmful environments such as dust. Despite self-administering oral cefixime (0.1 g twice daily for 2 days) and receiving subsequent intravenous ceftriaxone (2 g once daily for 2 days) for symptomatic treatment at a local hospital, his fever remained poorly controlled, with a maximum temperature (Tmax) of 40 °C. His past medical history included surgical treatment for tuberculous hydropneumothorax. It was confirmed that the standard first-line anti-tuberculosis regimen was administered postoperatively, and the treatment had been completed with a cure. Initial vital signs were: T 38.8 °C, HR 119 beats/min, BP 104/72 mmHg, SpO₂ 95%, and R 22 breaths/min. Physical examination revealed tachypnea and coarse breath sounds, with no significant dry or moist rales audible. Other physical findings were unremarkable. A subsequent chest computed tomography (CT) scan revealed a spherical, high-density opacity in the right lower lobe (Figures 1A,D). Routine blood tests revealed a decreased lymphocyte count (1.01 × 10^9^/L) and an elevated monocyte count (0.69 × 10^9^/L). The white blood cell count (5.61 × 10^9^/L), neutrophil count (3.90 × 10^9^/L), and platelet count (194 × 10^9^/L) were all within normal ranges. C-reactive protein (109 mg/L) and procalcitonin (0.21 ng/mL) were elevated, and hemoglobin was mildly decreased (125 g/L). Tests for rheumatology panel, cardiac enzymes, liver and kidney function, *Chlamydia pneumoniae* IgM, *Mycoplasma pneumoniae* IgM, T-SPOT, blood cultures, fungal markers (including (1,3)-β-D-glucan and galactomannan antigen, nucleic acid tests for influenza A virus, influenza B virus, rhinovirus, adenovirus, respiratory syncytial virus and SARS-CoV-2, as well as sputum culture, all returned negative results). Pulmonary function tests indicated mild restrictive ventilatory dysfunction and mild small airway obstruction. After admission, empirical antimicrobial therapy with moxifloxacin 0.4 g once daily, combined with piperacillin-tazobactam 4.5 g every 8 h for 3 days, was initiated. However, the patient continued to experience persistent fever, with a maximum temperature of 39 °C. Considering the patient’s history and poor response to conventional antibiotics, a high suspicion for infection with atypical pathogens, including *Cryptococcus* or *Chlamydia psittaci*, was raised. To further clarify the diagnosis, bronchoscopy was performed on the third day after the patient’s admission to the hospital. Subsequently, the patient underwent transbronchial lung biopsy (TBLB) of the posterior segment of the right lower lobe, guided by radial endobronchial ultrasound (r-EBUS). Biopsy samples were collected from the hypoechoic lesion, and bronchoalveolar lavage (BAL) was performed at the same site after the biopsy. The examination revealed mild congestion of the bronchial mucosa in the right lower lobe posterior segment, with a smooth mucosal surface and no signs of tumor growth or active bleeding. On hospital day 4, targeted next-generation sequencing (tNGS) of the bronchoalveolar lavage fluid (BALF) detected *Chlamydia psittaci*, with a normalized read count of 9,956. *Mycobacterium tuberculosis* was not detected, and no other potential pathogens were identified. Additionally, bacterial and fungal smears and cultures performed on the BALF sample all returned negative results. Based on the etiological findings, a diagnosis of *Chlamydia psittaci* pneumonia was confirmed. The piperacillin-tazobactam regimen was discontinued and switched to oral minocycline 0.1 g every 12 h. The patient’s body temperature returned to normal 3 days after starting the new treatment, and his cough became very occasional, with no dyspnea, indicating marked clinical improvement. The pathology report returned on hospital day 6 showed numerous acute and chronic inflammatory cells along with a small number of respiratory epithelial cells in the specimen from the right lower lobe posterior segment. Special stains for acid-fast bacilli, periodic acid-schiff stain (PAS), and methenamine silver were all negative, thereby ruling out pulmonary malignancy (Figure 2). A follow-up chest CT scan on day 10 of treatment showed significant resolution of the lesion compared to previous imaging (Figures 1B,E). The patient was subsequently discharged. A one-month follow-up chest CT scan demonstrated that the pulmonary inflammation had mostly resolved (Figures 1C,F), leaving only a faint, round hazy shadow. The patient has reported no specific discomfort during follow-up to date.

**Figure 1:**
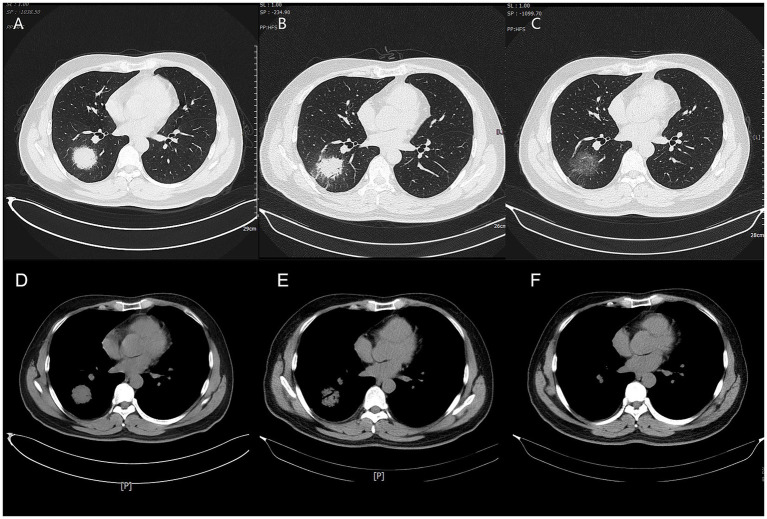
The patient’s chest CT images from day 3 of fever (February 16, 2025; **A**), day 10 of treatment (February 26, 2025; **B**), and day 32 of treatment (March 20, 2025; **C**). Image **(A)** reveals a spherical high-density shadow in the right lower lobe. Image **(B)** indicates that the exudation in the right lower lobe has been partially absorbed compared to the previous state. In image **(C)**, most of the exudate has resolved, leaving only mild haziness. Panels **D**–**F** correspond to Panels **A**–**C**, respectively, representing the mediastinal window views at the corresponding anatomical levels. This allows for comparison of the lesion characteristics in both lung and mediastinal windows.

**Figure 2:**
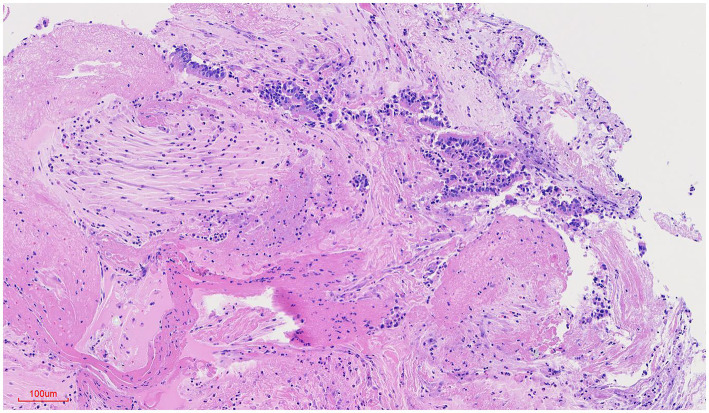
Transbronchial lung biopsy (hematoxylin and eosin staining) showed: examination of the right lower lobe posterior segment revealed numerous acute and chronic inflammatory cells, along with a small number of respiratory epithelial cells. Special stains: acid-fast (−), PAS (−), methenamine silver (−).

## 3 Discussion

This case involves a middle-aged male patient who presented with high fever. A CT scan revealed a spherical high-density shadow in the right lung. *Chlamydia psittaci* was detected in the bronchoalveolar lavage fluid by targeted next-generation sequencing (tNGS), confirming the diagnosis of spherical pneumonia caused by *Chlamydia psittaci* infection. After 21 days of treatment with 0.1 g of minocycline every 12 h, follow-up imaging showed significant resolution of the lesion. This case further expands the known spectrum of pathogens causing spherical pneumonia. It suggests that for patients with unexplained spherical pneumonia accompanied by signs of infection and a relevant occupational exposure history, such as frequent contact with birds (e.g., pet bird owners, pet store or poultry processing plant workers), atypical pathogens, particularly *Chlamydia psittaci*, should be considered. *Chlamydia psittaci* is a Gram-negative obligate intracellular pathogenic microorganism belonging to the genus *Chlamydia* within the family Chlamydiaceae (2). It is a pathogen associated with poultry and pet birds. *Chlamydia psittaci* exhibits a unique biphasic developmental cycle, comprising two forms: elementary bodies and reticulate bodies (13). It adapts to a wide range of hosts, including mammals and birds, and is primarily transmitted from birds to humans (14). While all bird species are susceptible, pet birds and poultry are the most common sources. Human infection with psittacosis can occur via direct exposure to infected animals, avian nasal secretions, infectious bird droppings, or inhalation of contaminated aerosols such as feather dust. Even brief contact with infected birds or contaminated environments can lead to human infection (15). Occasional human-to-human transmission has also been reported. For instance, during an outbreak in Sweden in 2013, a severely ill patient transmitted the infection to 10 secondary cases (16). Furthermore, a 2020 outbreak in China not only confirmed transmission between infected individuals and their close contacts but also observed secondary and tertiary transmission (17). After the pathogen is inhaled, *Chlamydia psittaci* first enters and proliferates within the reticuloendothelial cells of the liver and spleen (18). It then spreads through the bloodstream to organs such as the lungs. The clinical severity varies from asymptomatic infection to systemic manifestations. Primary symptoms after infection include fever, cough, dyspnea, headache, and myalgia, and it can be accompanied by severe pneumonia (19). Symptoms usually appear 5–14 days after exposure to the pathogen (15). The severity of the disease is related to factors such as the pathogen load, strain virulence, and the host’s immune status. The most common manifestations are fever, chills, severe headache, myalgia, malaise, and a non-productive cough. The clinical presentation of psittacosis is similar to that of community-acquired pneumonia caused by other pathogens. Common laboratory findings include a normal white blood cell count with elevated neutrophil percentage and lymphocytopenia during the acute phase, along with markedly elevated inflammatory markers (CRP, ESR, PCT, IL-6) (20). Additionally, some patients present with elevated liver enzymes (AST and ALT). Radiologically, lobar consolidation is the most frequently reported pattern. Extrapulmonary manifestations can involve the heart, liver, skin, and central nervous system (21, 22). Other reported complications include endocarditis, myocarditis, pericarditis, interstitial nephritis, acute renal failure, hepatosplenomegaly, rose spots, erythema multiforme, and erythema nodosum (23). Our patient exhibited several typical features: acute onset, high fever (≥39 °C), myalgia, lymphocytopenia, a normal white blood cell count, and markedly elevated CRP and PCT. Notably, the patient did not show the more common radiological pattern of lobar pneumonia. Instead, chest CT revealed the less frequently reported presentation of spherical pneumonia. Furthermore, typical markers of hepatic involvement were also absent. Without timely diagnosis or treatment, psittacosis pneumonia can rapidly progress to severe pneumonia and even lead to death (24–26). Studies have indicated that CRP, PCT, and arterial partial pressure of oxygen can help differentiate severe cases from non-severe ones (27). The literature also suggests that the number of mNGS sequences may be used to assess disease severity and prognosis (28).

However, over the past few decades, reports of this pathogen have been limited due to diagnostic constraints. *Chlamydia psittaci* is reported to account for approximately 1–2% of community-acquired pneumonia cases (29, 30). This figure likely underestimates the true incidence, as routine microbiological testing does not typically include *Chlamydia psittaci* detection (31). Furthermore, traditional culture methods are technically challenging and time-consuming (taking up to several weeks). Meanwhile, serological antibody testing requires paired acute and convalescent samples, leading to delayed confirmation. Additionally, antibiotic use can delay or attenuate the antibody response, affecting test accuracy. tNGS is a next-generation sequencing (NGS) method that detects a predetermined range of target genes. With the widespread application of NGS technology (32), the detection rate of *Chlamydia psittaci* infections is increasing significantly. A literature review analyzed 54 cases of severe *Chlamydia psittaci* pneumonia, 12 of which were diagnosed via mNGS (20). As a faster and more cost-effective molecular detection method compared to mNGS, targeted next-generation sequencing (tNGS) can shorten the time to diagnosis to 2–4 days (33), which is significantly faster than the previously reported 5–11 days (34). In this case, the diagnosis was confirmed by tNGS on the fourth day of hospitalization, demonstrating its advantage in rapid pathogen identification. The detection of *Chlamydia psittaci* by tNGS in this case was considered clinically significant based on the following content (35): a high number of specific sequence reads uniquely mapped to the pathogen; and, most importantly, a strong correlation with the patient’s epidemiological history (bird exposure). We note that the interpretation of tNGS requires integrating microbiological data with the clinical context to distinguish true infection from colonization or background noise. Compared to conventional PCR and serology, next-generation sequencing (NGS) is particularly suitable for diagnosing complex, rare, or mixed infections.

Common imaging manifestations of *Chlamydia psittaci* infection include consolidation (36), solitary nodules, and ground-glass opacities (37, 38). Some patients may present with mediastinal or hilar lymphadenopathy. Lesions are often unilateral, but they can involve multiple lobes or even both lungs, sometimes accompanied by interstitial changes (39). The halo sign and reversed halo sign (RHS) are less common, and presentation as spherical pneumonia is even rarer. It has been reported in previous cases that *Chlamydia psittaci* infection can present with imaging features of spherical pneumonia on X-ray (9). Yang et al. (40) demonstrated that psittacosis pneumonia primarily manifests in two imaging patterns: lobar pneumonia and spherical pneumonia types with interstitial changes, and the former is more common. Three key radiographic signs—intralobular lines, air bronchogram sign, and reverse halo sign—indicate simultaneous involvement of the parenchyma and interstitium. It outlines five stages of CT follow-up in psittacosis pneumonia. In the very early stage, imaging reveals vascular inflammatory changes, typically manifesting as ground-glass opacities around small core vessels or thickening of hilar vessels. In the early stage, secondary pulmonary lobules appear as high-density shadows. The progressive stage is characterized by rapid consolidation into large areas, easily evolving into lobar pneumonia. The repair and resolution stages tend to form a central reverse halo sign, while the dissipation stage may lead to the formation of fibrous bands.

Round pneumonia is a type of pneumonia or pulmonary infection that appears as a round shadow on chest X-ray or CT (41, 42). It was first reported by Greenfield and Gyepes (43). The current view is that it results from the spherical spread of inflammatory exudate along the bronchi or alveolar pores following an acute viral or bacterial infection. Some scholars believe this diffusion is a rare and specific form of pulmonary inflammation caused by centrifugal and equidistant spread. Radiologically, it most commonly manifests as a round or round-like mass shadow. In radiological studies, round pneumonia can present as a nodule or mass up to 7 cm in diameter, often located in the lower and posterior lobes near the pleura. It may exhibit an air bronchogram sign, with smooth or ill-defined margins, and satellite lesions can also be present. Spherical pneumonia is generally more prevalent in children. Only around 1% of round pneumonia cases occur in adults (44). The exact mechanism remains unclear, but it may be related to the following factors: First, children have underdeveloped alveolar connections (pores of Kohn, channels of Lambert), tighter connective tissue septa, and smaller alveoli (45). After pathogenic bacteria invade the lung tissue and trigger an inflammatory response, exudate spreads through the alveolar pores, forming inflammatory lesions of approximately equal diameter (41). The adult’s immune response might have been robust enough to prevent lobar or diffuse spread but not rapid enough to eliminate the infection quickly, resulting in a circumscribed inflammatory mass. These lesions are often distributed in the peripheral lung fields near the pleura, resulting in well-defined spherical consolidations. Second, certain pathogens exhibit a strong affinity for alveolar epithelial cells. For example, infections caused by *Streptococcus pneumoniae* are more likely to present as spherical pneumonia on imaging compared to other pathogens (46). Our case demonstrates that *Chlamydia psittaci* infection can also manifest as spherical pneumonia on imaging. Third, the timing of the disease plays a role: imaging findings represent a snapshot at a specific stage. Considering our patient’s early presentation, the imaging may reflect the initial centrifugal spread of inflammation. Based on the patient’s chest CT findings showing a round or round-like lesion, the differential diagnosis must cover all diseases that present as round or quasi-round opacities on imaging. Non-infectious etiologies primarily include pulmonary tumors, focal organizing pneumonia, immune-related diseases [e.g., Wegener’s granulomatosis, rheumatoid nodules (47)], pulmonary vascular lesions (e.g., pulmonary arteriovenous fistula, hemangioma), and rounded atelectasis. Infectious etiologies mainly include granulomatous infections (e.g., *Mycobacterium tuberculosis*, Aspergillus infection), typical pathogen infections [such as *Streptococcus pneumoniae* (46)*, Klebsiella pneumoniae* (48) and *Pseudomonas aeruginosa* (12)], atypical pathogen infections [e.g., *Coxiella burnetii* (49)], and viral infections [e.g., coronavirus (50)]. There has been a case report of pulmonary actinomycosis that was misdiagnosed as lung cancer, which led to a right upper lobectomy. The diagnosis of pulmonary actinomycosis was only confirmed by postoperative pathology (51). For these reasons, obtaining a definite histological diagnosis via transbronchial lung biopsy (TBLB) was essential. Pathological examination detected no caseous necrosis or tumor cells. Finally, targeted next-generation sequencing (tNGS) performed on the patient’s bronchoalveolar lavage fluid returned a positive result for *Chlamydia psittaci*. In recent years, with the widespread application of NGS technology (33, 52), the diagnostic rate for the etiology of complex pulmonary infections has significantly improved, offering new possibilities for precise and individualized treatment. Particularly in the context of immunodeficiency or multidrug resistance, NGS demonstrates its irreplaceable value, enabling clinicians to rapidly identify the causative pathogen. This helps shorten the duration of empiric broad-spectrum antibiotic use, thereby reducing the risk of drug resistance and the incidence of adverse drug reactions.

Due to the absence of a cell wall in *Chlamydia psittaci*, β-lactam antibiotics are ineffective against it, and their empirical use should be avoided in clinical practice. Current treatment primarily involves tetracyclines, macrolides, and fluoroquinolones, with tetracyclines being the first-line choice. Examples include doxycycline, minocycline, and the newer tetracycline agent omadacycline, tigecycline. Omadacycline exerts its antibacterial effect by binding to the bacterial 30S ribosomal subunit, thereby inhibiting protein synthesis. It is not metabolized by the liver or kidneys. Several studies have demonstrated its favorable efficacy in the treatment of psittacosis (53–55). Cases have been reported in which tigecycline was successfully used to treat severe psittacosis pneumonia (20). However, whether tigecycline is superior to other tetracyclines in severe patients requires further large-scale clinical observation. When tetracyclines are contraindicated, such as in pregnant women, children, or patients with allergies, macrolides like erythromycin or azithromycin are recommended. Due to relatively high resistance rates in recent years, fluoroquinolones are not recommended as first-line treatment for psittacosis but can serve as a second-line option, particularly for patients intolerant to tetracyclines or macrolides. In this case, the patient was in generally good condition, so minocycline was selected and proved effective.

## 4 Conclusion

The clinical symptoms of *Chlamydia psittaci* pneumonia lack specificity. Therefore, clinicians need to establish a comprehensive diagnostic approach that integrates “clinical presentation, imaging findings, and etiological evidence.” It is crucial to emphasize the collection of epidemiological history, including exposure to birds, visits to live poultry markets, and occupational risk factors. These clues are of great value in narrowing the differential diagnosis. In terms of treatment strategy, once *Chlamydia psittaci* infection is suspected, it is recommended to perform NGS for etiological confirmation as early as possible, if available, to achieve a precise diagnosis. However, considering the variations in medical resources and cost across different regions, strengthening epidemiological assessment and combining it with clinically accessible laboratory testing methods, such as serology and PCR, serves as an important alternative approach when NGS is not immediately available. Once diagnosed, effective antibiotic therapy should be initiated promptly after a comprehensive assessment of the patient’s condition. Early recognition and intervention can reduce the incidence of severe psittacosis pneumonia and improve the prognosis.

## Data Availability

The raw data supporting the conclusions of this article will be made available by the authors, without undue reservation.
