# Supplementary figures and images for: A case report of spherical pneumonia caused by Chlamydia psittaci infection
Source: Front Med (Lausanne). 2026 Jun 8;13:1841775. doi: 10.3389/fmed.2026.1841775 (PMC13283970; doi:10.3389/fmed.2026.1841775)

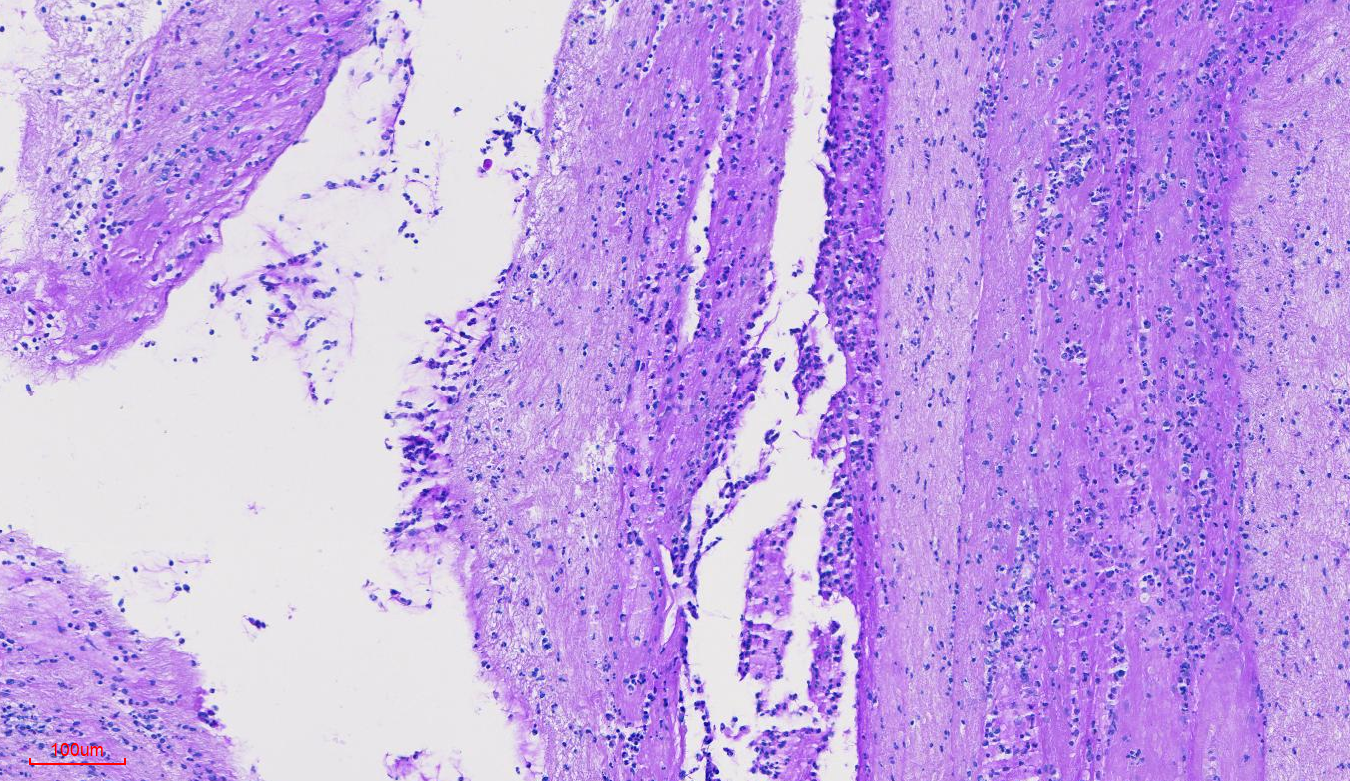

Supplement: Supplementary file 1 [file Image_1.PNG]

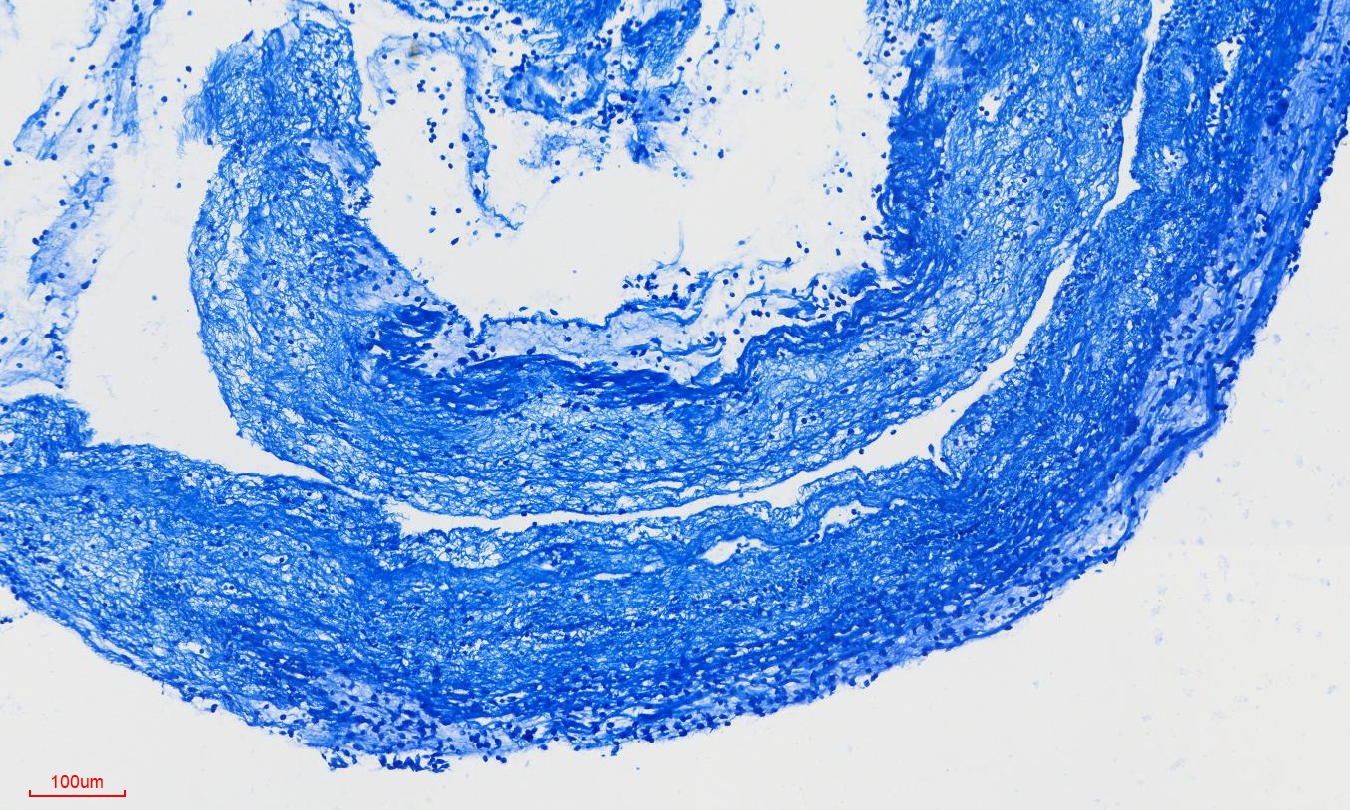

Supplement: Supplementary file 2 [file Image_2.PNG]

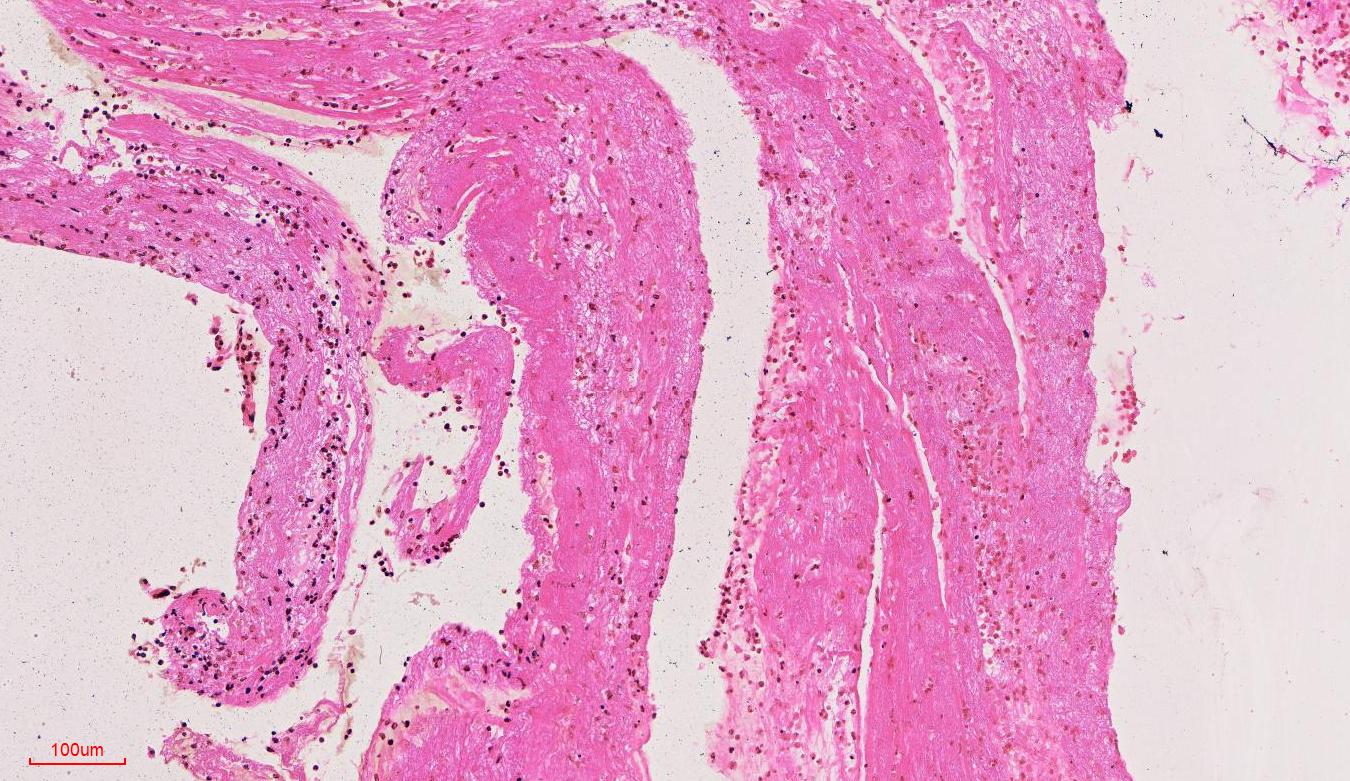

Supplement: Supplementary file 3 [file Image_3.PNG]
